# TiO_2_ nanoparticles: Green synthesis and their role in lessening the damage of *Colletotrichum graminicola* in sorghum

**DOI:** 10.1002/fsn3.4297

**Published:** 2024-07-22

**Authors:** Ghulam Nabi, Tehmina Anjum, Zill‐e‐Huma Aftab, Humaira Rizwana, Waheed Akram

**Affiliations:** ^1^ Department of Plant Pathology, Faculty of Agricultural Sciences University of the Punjab Lahore Pakistan; ^2^ Department of Botany and Microbiology, College of Science King Saud University Riyadh Saudi Arabia; ^3^ Vegetable Research Institute Guangdong Academy of Agricultural Sciences Guangzhou China

**Keywords:** *Colletotrichum graminicola*, green synthesis, nanoparticles, sorghum, titanium dioxide

## Abstract

Fungal pathogens pose a persistent threat to crop plants, risking global food security. Anthracnose of sorghum caused by *Colletotrichum graminicola* causes a considerable loss in sorghum production. This study aimed to manage the anthracnose disease in sorghum using green‐synthesized TiO_2_ nanoparticles using pomegranate peel and to assess their impacts on the agroeconomic attributes of sorghum. Synthesized TiO_2_ nanoparticles showed strong dose‐dependent antifungal activity against *C. graminicola* and significantly reduced mycelial radial growth, comparable to commercial fungicides. Foliar application of TiO_2_ at concentrations of 150 and 200 ppm reduced the disease index >60% in pot trials. Additionally, the effect of TiO_2_ NPs on the growth and yield of sorghum plants and the possible mechanism(s) behind the suppression of anthracnose disease were deciphered. TiO_2_ NPs also improved shoot and root length, biomass accumulation, penile size, number of grains, and grain weight in sorghum plants infected with *C. graminicola*. Application of TiO_2_ NPs significantly increased the content of defense‐related biochemicals, including total phenolic contents, activities of defense‐related enzymes (PO, PPO, and PAL), photosynthetic pigments (total chlorophyll contents and carotenoids), and total protein contents. Collectively, our study verified the potential of green‐synthesized titanium dioxide nanoparticles to suppress anthracnose disease by activating a defense system and stimulating growth and yield promotion under pathogen stress.

## Graphical abstract

Collectively, this study verified the potential of green‐synthesized titanium dioxide nanoparticles to suppress anthracnose disease by activation of a defense system and simulations growth‐ and yield promotion under pathogen stress.

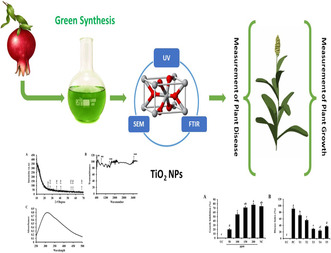

## 1 INTRODUCTION

Sorghum (*Sorghum bicolor* [L.] Moench) is an important cereal and fodder crop cultivated worldwide. In many countries, particularly in Africa, Asia, and some areas of the Americas, sorghum has great cultural and economic significance. Due to its great adaptability, sorghum can grow in regions with poor soil and little rainfall. Its resilience makes it a valuable crop in semi‐arid and dry areas where other cereal crops would find it difficult to flourish (Yadav et al., 2023). This cereal crop continues to play a significant role in global agriculture, contributing to sustainable farming methods and food security. *Colletotrichum graminicola* is among various plant pathogens that damage the sorghum crop and cause significant yield losses (Hassan et al., 2021). This fungal pathogen causes red leaf spots and black anthracnose spots on the above ground parts of sorghum plants. Due to its wide distribution and ability to damage all aerial parts of the plant, anthracnose is one of the most important diseases of the sorghum crop (Abreha et al., 2021).

Nanomaterials (NMs) are molecules that range in diameter from 1 to 100 nm and play an important role in plant development and productivity, especially under stress conditions (Sarraf et al., 2022). These nanoparticles also function as fertilizers, lowering fertilizer input while minimizing nutrient loss. Titanium dioxide nanoparticles (NPs) are widely used in a variety of fields, such as energy, medicine, and basic research (Irshad et al., 2021). The exact process through which titanium dioxide NPs impact plant growth and development is unknown. Studies have linked the reactive oxygen species (ROS) pathway to plant responsiveness to titanium dioxide (Cox et al., 2016). By introducing green nanoparticle production, which has proven to be highly successful, scientists are trying to lessen the detrimental impacts of nanoparticles made by chemical techniques (Duan et al., 2015). Since the chemicals utilized in the process are usually toxic and flammable, chemically synthesized nanomaterials are not ecologically friendly (Dikshit et al., 2021). Applications of titanium‐based nanomaterials in agriculture have attracted a lot of attention (Javed et al., 2022). Titanium dioxide NPs are being used by researchers to enhance crop productivity. The physicochemical characteristics of the soil were enhanced by a soil‐based titanium dioxide treatment (Asadishad et al., 2018). Moreover, titanium dioxide improved plant growth and productivity in *Silybum marianum*, as well as the relative water content (RWC) and chlorophyll content (Jafari et al., 2023).

In this study, we green‐synthesized titanium dioxide nanoparticles using pomegranate peel extract. We analyzed the effect of these nanoparticles on the growth of *C. graminicola* and the severity of anthracnose disease caused by *C. graminicola* in sorghum plants. The effect of TiO_2_ NPs on the plant defense activation, growth, and yield attributes of Sorghum was also examined.

## 2 MATERIALS AND METHODS

### 2.1 Reagents and chemicals

Titanium tetraisopropoxide and sodium hydroxide were purchased from Sigma‐Aldrich. Pomegranate peel was collected from the fruit juice shops situated in the market of Punjab University, Lahore, Pakistan.

### 2.2 Preparation of *Punica granatum* extract

Pomegranate peel was chopped into small pieces, dried, and ground into a powder. To prepare the extract, 15 g of powder was mixed with 150 mL of double‐distilled water and agitated for 20 min at 90°C and 900 rpm. Afterward, the extract was filtered using Whatman No. 1 filter paper. The clear peel extract was stored at 4°C until further use.

### 2.3 Preparation of titanium dioxide nanoparticles

A 0.2 M solution of titanium tetraisopropoxide was prepared in distilled, sterilized water. The 50 mL extract of *P. granatum* was added to a 0.2 M solution of titanium isopropoxide at a 1:1 ratio for the green synthesis of titanium dioxide nanoparticles. The mixture was stirred by a magnetic stirrer at a rate of 6000 rpm for 1 h. Following this, the mixture was centrifuged at 4000 rpm for 20 min. The pellet was collected and washed to remove the impurities. The pH of the solution was adjusted to 7. The solution was again centrifuged at 4000 rpm for 20 min after washing the pellet. The pellet was collected, mixed with distilled, sterilized water and placed into the hot air oven at 150°C for 2 h. After complete drying, the pellet was then converted into a fine powder. The fine nanoparticle powder was stored at room temperature in the lab for further use.

### 2.4 Characterization of nanoparticles

On Rigaku 600Miniflex X‐ray diffraction (XRD) equipment, the X‐ray diffraction investigations of titanium dioxide NPs were carried out using copper k‐ray radiation (*λ* = 1.5412) in the scanning range of 100–800. Agilent Technologies Cary 60 UV–vis was used to obtain UV–visible (UV–vis) spectra in the wavelength range of 200–600 nm to confirm the absorbance of titanium dioxide NPs and detect changes in absorbance brought on by changes in reaction situations. To detect the distinctive functional groups, present on the surface of the titanium dioxide, Fourier transform infrared (FTIR) spectra of all samples were taken in the 650–4000 cm^−1^ range. Titanium dioxide nanoparticles synthesized in green were morphologically characterized using high‐resolution scanning electron microscopy (HR‐SEM).

### 2.5 In‐vitro antifungal activity of nanoparticles

*Colletotrichum graminicola* isolate was obtained from the First Fungal Culture Bank of Pakistan, University of the Punjab, Lahore, Pakistan, previously isolated from Sorghum bicolor having leaf spot disease. The food poisoning technique was used to investigate the antifungal activity of TiO_2_ against *C. graminicola*. As the NP structures were stable up to 350°C, the nanoparticles were added to the 2% Potato Dextrose Agar (PDA) media prior to autoclaving. TiO_2_ NPs were mixed in the media at concentrations of 50, 100, 150, and 200 ppm. A disc of fungal material from a nine‐day‐old culture was placed in the media plates as inoculum. Media plates lacking NPs were used as a control. The inoculated media plates were kept in an incubator for 7 days at 25 ± 2°C. The fungal growth inhibition was calculated using the formula shown below (Yazid et al., 2023).

$$\text{Inhibition}\;\left(\%\right)=\frac{d_{c}-d_{t}}{d_{c}}100$$

where *d*_c_ is the mean diameter of the growth in the control and *d*_t_ is the average growth diameter in the treatment. Three replicates of each treatment were set in each trial.

### 2.6 The potential of titanium dioxide NPs to manage *Colletotrichum* leaf spots in sorghum

#### 2.6.1 Preparation of the fungal inoculum

*Colletotrichum graminicola* culture was grown on PDA medium under continuous light at 35°C. After 8–10 days of incubation, 10 mL of distilled autoclaved water was poured onto the media plate, and fungal mycelia and spores were gently scratched. The concentration of the suspensions was adjusted to 10^5^ conidia/mL with the help of a hemocytometer.

#### 2.6.2 Pot experiment

The seeds of the sorghum variety “HSG‐030C” were purchased from the Growtech Seeds Company (Sahiwal, Pakistan). Plants were raised in plastic pots filled with sterilized sandy loamy soil. The aqueous solutions of NPs at different concentrations were prepared by sonicating the titanium dioxide NPs. Details of treatments are mentioned in Table 1. One month after emergence, the spore suspension of the pathogen was injected into the midrib of the plants using the sterilized syringe. The next day, the plants were sprayed with the aqueous solutions of NPs. Copper oxychloride @ 2.5 g/L was sprayed as a fungicide control (Iliger et al., 2021; Jasrotia et al., 2021). Plants were kept in the greenhouse for incubation under natural daylight conditions. Plants were provided with distilled, sterilized water when needed. Five replicate plants were included in each treatment.

**TABLE 1:** Details of treatments.

Treatments	Code
Untreated control	UC
Pathogen only	PC
TO_2_ NPs @ 50 mg/L + Pathogen	T1
TO_2_ NPs @ 100 mg/L + Pathogen	T2
TO_2_ NPs @ 150 mg/L + Pathogen	T3
TO_2_ NPs @ 200 mg/L + Pathogen	T4
Copper oxychloride @ 2.5 g/L + Pathogen	T5

#### 2.6.3 Evaluation of the disease index

Disease assessments were performed 30 days post‐inoculation. Anthracnose infection response was evaluated using a 1–5 rating scale [0 = No symptoms, 1 = 5% stripes on the leaves, 2 = 6%–20% stripes on the leaves, 3 = 21%–40% stripes on the leaves, 4 = 41%–60% stripes on the leaves, 5 = >61% stripes on the leaves] based on disease response observed on inoculated leaves. The disease index and disease index in percentage were calculated using the formula (Iftikhar et al., 2006):

$$\begin{array}{c} \text{Disease index}=\left(\text{Stripes in scale}\;\mathrm{one}\right)+\left(\text{Stripes in scale}\;\mathrm{two}\right)\\ \;+\left(\text{Stripes in the scale five}\right) \end{array}$$

$$\text{Disease index}\;\left(\%\right)=\frac{\text{Disease index}}{\text{Total infected plants}}\times \frac{100}{5}$$

### 2.7 Quantification of defense‐related biochemicals

#### 2.7.1 Quantification of total phenolics

Quantification of total phenolic contents was performed using the Folin–Ciocalteu reagent method (Singleton & Rossi, 1965). One hundred mg of fresh leaf material was homogenized in 1 mL of methanol. The mixture was centrifuged to obtain a clear solution. About 200 μL of the prepared solution was mixed with 0.1 mL of 50% Folin–Ciocalteu reagent and 1.2 mL of distilled water. The mixture was incubated for 5 min. Afterward, 0.5 mL of 20% (w/v) sodium carbonate solution was added. The absorbance was recorded at 766 nm. The gallic acid standard curve was prepared, and total phenolic contents were expressed as μg/g/FW.

#### 2.7.2 Quantification of defense‐related enzymes

The representative leaf samples were collected from sorghum plants. One gram of the plant sample was crushed in 2 mL of 0.1 M phosphate buffer (pH 7) in a pre‐chilled pestle and mortar. The homogenate was centrifuged at 12,000 rpm at 4°C for 10 min, and the obtained supernatant was used as crude enzyme extract.

Peroxidase activity was measured using the Bateman method (Bateman, 1967). The enzyme extract (0.2 mL) was mixed with 5 mL of (M) pyrogallol prepared in 0.005 M phosphate buffer (pH 6). The reaction was stopped by adding 0.5 mL of 1% H_2_O_2_. Changes in absorbance were observed at 420 nm. For PPO quantification, the enzyme mixture consisted of 2.6 mL of phosphate buffer (pH 6.5), 0.1 mL of L‐3,4‐dihydroxyphenylalanine (L‐DOPA) 5 mM, 0.1 mL ascorbic acid (2.1 mM) and 0.1 mL of crude enzyme extract. The whole mixture was incubated for 10 min at room temperature. The OD was then measured at 265 (Karthikeyan et al., 2006). PAL activity was measured by the method of Sainders and McClure (1975). The reaction mixture consisted of 200 μL of enzyme extract, 200 mM TrisHCl (pH 7.0) as a buffer solution, and 20 mM l‐phenylalanine. The whole mixture was incubated for an hour at room temperature, and OD was measured at A290 nm. The enzyme activities were expressed as μmol/min/g.

### 2.8 Evaluation of growth attributes

Plants were carefully uprooted from the pots after 75 days of nanoparticle application. Shoot length and root length were measured and expressed in cm. Afterward, fresh and dry biomasses were quantified. The dry biomass was quantified by keeping plant samples in the oven at 60°C for 5 days.

### 2.9 Analysis of the physiological parameters

#### 2.9.1 Quantification of total chlorophyll and carotenoid contents

One gram of fresh leaves was ground with 15 mL acetone. Afterward, the solution was filtered, and the absorbance was observed at 645 and 663 nm. The estimation of total chlorophyll and carotenoid contents was determined using the following equations (Arnon, 1949; Davies & Goodwin, 1976).

$$\text{Total chlorophyl contents}\left(\frac{\mathrm{mg}}{g}\right)=\left(D645\times \frac{1000}{34.5}\right)$$

$$\text{Carotenoid contents}\left(\frac{\mathrm{mg}}{g}\right)=\mathrm{OD}480+\left(0.114\times \mathrm{OD}663\right)―\left(0.638\times \mathrm{OD}645\right)$$

#### 2.9.2 Estimation of total protein content

The total protein content of leaves was measured by the Bradford method (Arora & Wisniewski, 1994). Plant leaves were homogenized in liquid nitrogen. The powder material was extracted with sodium phosphate buffer (50 mM, pH 7.6) and centrifuged at 14,000 *g* for 10 min at 4°C. The OD of the supernatant was measured at 532.

### 2.10 Statistical analysis

All the data were analyzed statistically by performing ANOVA and DNMRT using the Excel add‐in “DSAASTAT” (Onofri & Pannacci, 2014). Correlation and PCA analysis were performed using Origin Pro software (Pro, 2022).

## 3 RESULTS

### 3.1 Characterization of nanoparticles

#### 3.1.1 X‐ray diffraction

**FIGURE 1:**
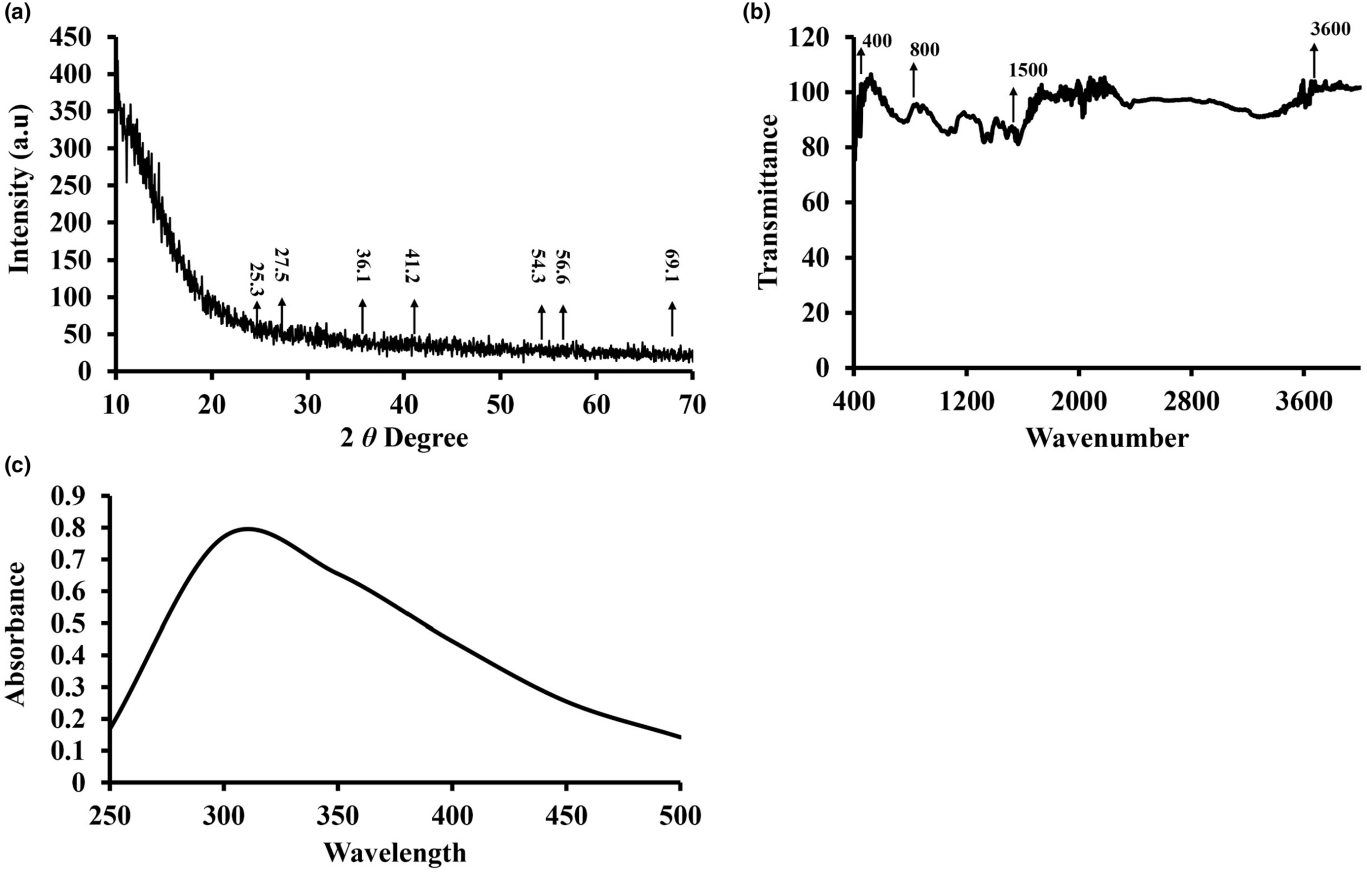
Characterization of titanium dioxide nanoparticles biosynthesized using *Punica granatum* peel extract. (a) X‐ray diffraction spectrum of titanium dioxide nanoparticles, (b) FTIR pattern of titanium dioxide nanoparticles, (c) UV–Vis spectra for titanium dioxide nanoparticles.

An XRD (X‐ray diffraction) graph of titanium dioxide nanoparticles (NPs) synthesized using pomegranate peel extract is interpreted by analyzing the diffraction peaks to identify the crystallographic phases, crystallite size, and other structural properties of the nanoparticles (Figure 1a). In the XRD graph, the peaks show X‐ray diffraction by the phases of crystals in the sample. Each peak is associated with a certain angle, which is connected to the interatomic gap inside the crystal lattice. When the peaks are compared to known values in the literature for titanium dioxide phases, the phases' common 2 values are about 25.3° and 27.5° for anatase and 36.1°, 41.2°, 54.3°, 56.6°, and 69.1° for rutile (Figure 1a).

#### 3.1.2 Fourier transform infrared

The FTIR study of titanium dioxide synthesized using pomegranate peel extract reveals important information about the chemical composition and interactions involved in nanoparticle production. The emergence of significant absorption bands in the 400–700 cm^−1^ range indicates the presence of Ti–O stretching vibrations, verifying the effective production of titanium dioxide (TiO_2_) nanoparticles (Figure 1b). These vibrations are caused by the bonds formed between titanium and oxygen atoms in the TiO_2_ crystal lattice. Peaks in the 1400–1600 cm^−1^ range indicate the existence of C=C and C–H stretching movements, which can be linked to organic chemicals found in pomegranate peel extract (Figure 1b). These organic compounds may have played a role in the titanium dioxide nanoparticles' stabilization and capping. The existence of O–H stretching vibrations on the surface of the titanium dioxide nanoparticles is indicated by the large absorption band at 3200–3600 cm^−1^. These groups of hydroxyls are most likely the result of interactions between the extract of pomegranate peel and the nanoparticles that were synthesized. Additional peaks in the 600–800 cm^−1^ range are compatible with Ti–O–Ti stretching movement, indicating the crystalline configuration of titanium and oxygen atoms inside TiO_2_ nanoparticles (Figure 1b). The crystal structure and bonding configuration of the synthesized nanoparticles are reflected in these vibrations.

In conclusion, the FTIR examination of titanium dioxide synthesized using pomegranate peel extract shows that TiO_2_ nanoparticles were successfully formed with contributions from organic components in the extract. The FTIR spectrum's different absorption bands reveal significant details about the chemical bonding, surface changes, and crystal arrangement of the synthesized nanoparticles.

#### 3.1.3 Ultraviolet–visible

The green titanium dioxide nanoparticles produced have UV–vis absorption spectra ranging from 250 to 600 nm (Figure 1c). Titanium dioxide nanoparticles' main absorption bands were observed at 338 nm. When exposed to a light source, titanium dioxide nanoparticles absorb light, causing electrons to be excited from the valence shell to the conduction band.

#### 3.1.4 Scanning electron microscopy

**FIGURE 2:**
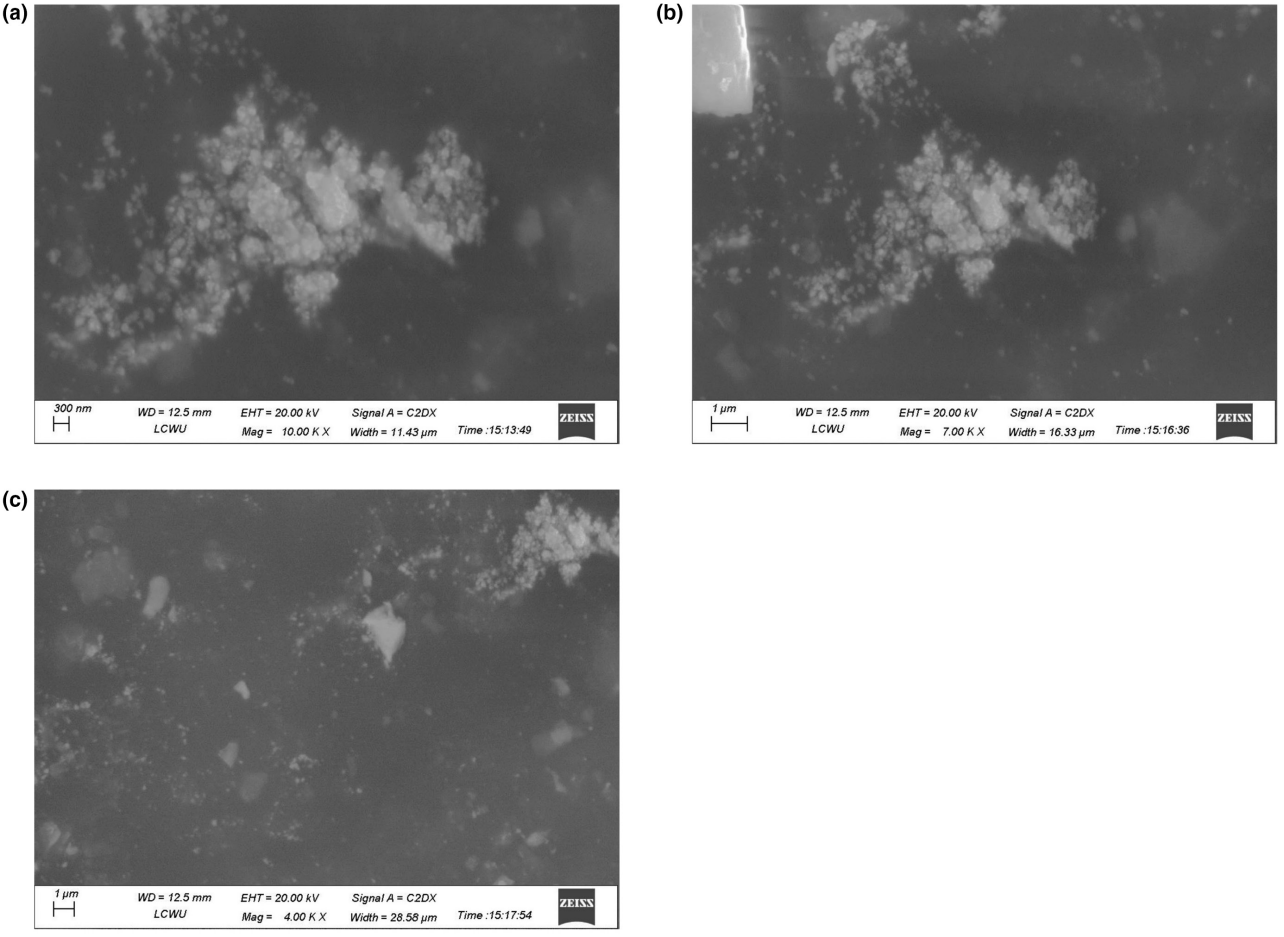
SEM image of titanium dioxide nanoparticles at various magnifications.

Titanium dioxide nanoparticles made from pomegranate peel extract were examined using SEM to learn more about their shape, size distribution, and surface properties (Figure 2). It was found that the synthesized nanoparticles were smaller in range (10–20 nm) and a greater size range was also obtained (100–200 nm).

### 3.2 Antimicrobial activity of green‐synthesized titanium dioxide NPs

**FIGURE 3:**
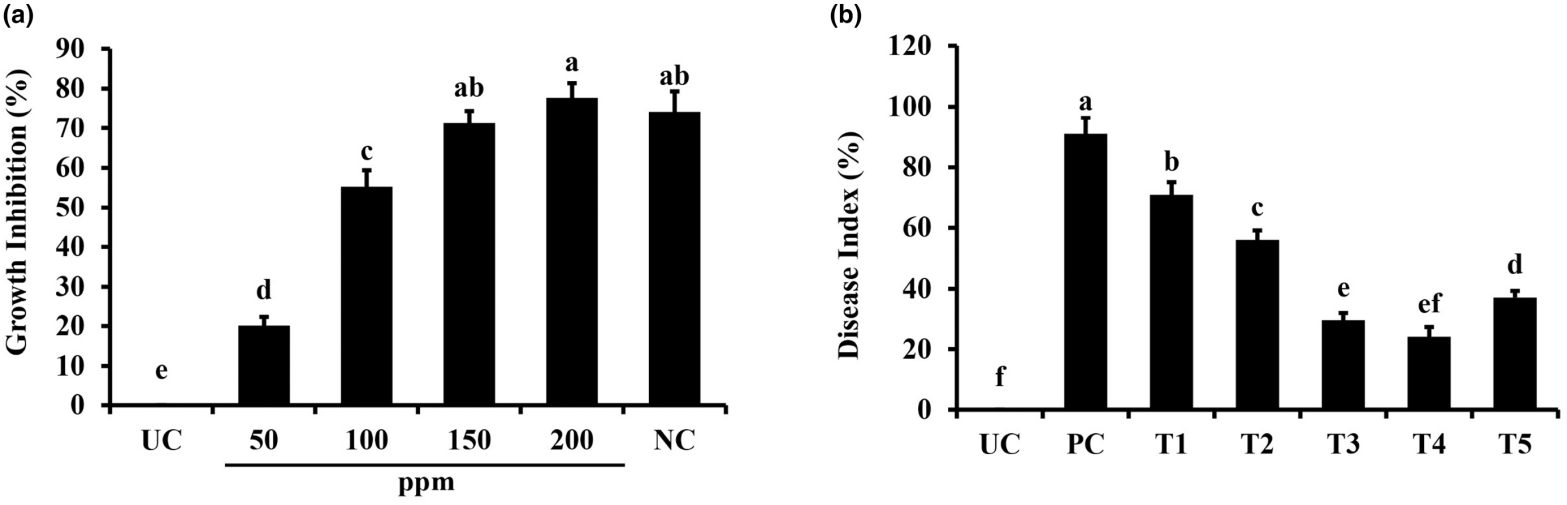
Bioactivities of titanium dioxide NPs. (a) Antifungal effects of biosynthesized titanium dioxide NPs against *Colletotrichum graminicola*. (b) Suppression of Colletotrichum leaf spot disease using titanium dioxide NPs. Vertical bars represent the standard error. Small letters represent the level of significance according to ANOVA and DNMRT (*p* > .05). PC, pathogen alone; T1, TiO_2_ NPs @ 50 ppm + Pathogen; T2, TiO_2_ NPs @ 100 ppm + Pathogen; T3, TiO_2_ NPs @ 150 ppm + Pathogen; T4, TiO_2_ NPs @ 200 ppm + Pathogen; T5, copper oxychloride @ 2.4 g/L + Pathogen; UC, untreated control.

The agar dilution assay was performed to evaluate the antifungal efficacy of green‐synthesized TiO_2_ NPs, which demonstrated their ability to inhibit *C. graminicola* strongly. With an increase in NP concentration, the percentage of inhibition has been observed to increase overall (Figure 3a). The lowest measured inhibition was 15.6% on Petri plates with 50 ppm TiO_2_ NPS (Figure 3a). When the NP concentration was raised to 150 ppm, there was a substantial increase in the inhibitory potential of 23.4%. When used at 2.5 g/L, copper oxychloride's inhibitory impact on fungal growth was found to be similar to the nanoparticle tested dose of 100 ppm, which inhibited fungal growth up to 77.67% (Figure 3a).

### 3.3 Effect of titanium dioxide NPs on suppression of anthracnose disease

Green‐synthesized titanium dioxide nanoparticles significantly reduced the disease index in treated plants (Figure 3b). TiO_2_ nanoparticles in concentrations of 150 and 200 ppm reduced the disease index up to 68.13% and 71.62%, respectively, compared to the pathogen control plants. The highest tested concentration of nanoparticles, i.e., 200 ppm, was found to be equally effective as that of the selected fungicide copper oxychloride (Figure 3b). TiO_2_ nanoparticles at lower concentrations reduced disease index up to 21.97% (TiO_2_ @ 50 ppm) and 36.46% (TiO_2_ @ 100 ppm) compared to pathogen control, respectively (Figure 3b).

### 3.4 Quantification of defense‐related biochemicals

**FIGURE 4:**
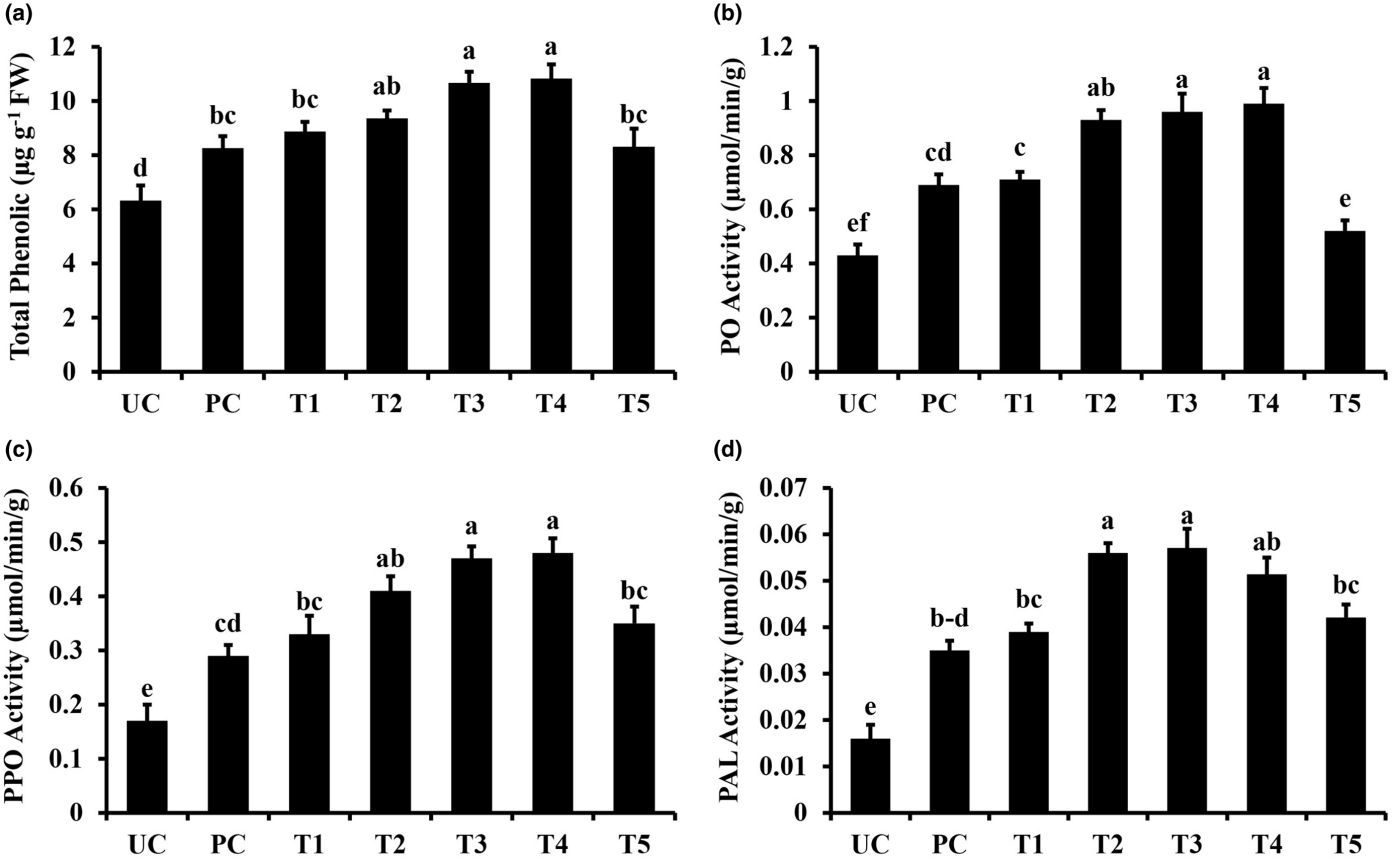
Effect of titanium dioxide NPs on defense‐related biochemicals of sorghum. (a) Total phenolic contents, (b) PO (Peroxidase) activity, (c) PPO (Polyphenol) activity, and (d) PAL (phenyl alanine ammonia lyase activity). Vertical bars represent the standard error. Small letters represent level of significance according to ANOVA and DNMRT (*p* > .05). PC, pathogen alone; T1, TiO_2_ NPs @ 50 ppm + Pathogen; T2, TiO_2_ NPs @ 100 ppm + Pathogen; T3, TiO_2_ NPs @ 150 ppm + Pathogen; T4, TiO_2_ NPs @ 200 ppm + Pathogen; T5, copper oxychloride @ 2.4 g/L + Pathogen; UC, untreated control.

The study showed that the inducible quantities of defense‐related biochemicals, including total phenolics and enzymes involved in the phenylpropanoid pathway, were increased by the application of TiO_2_ NPs (Figure 4). Total phenolic contents were significantly increased under treatment with TiO_2_ NPs compared to the pathogen control plants (Figure 4a). Treatments T3 (NPs 150 ppm + Pathogen) and T4 (NPs 200 ppm + Pathogen) increased total phenolic content up to 29.37% and 33.09%, respectively, compared to the pathogen control plants (Figure 4a).

Similarly, there was a significant difference in the quantities of PO, PPO, and PAL between NPs inoculated and noninoculated plants (Figure 4b–d). Relatively less difference was seen between pathogen control and non‐treated control plants. The treatments T3 and T4 increased PO and PPO contents up to 31.19%, 37.26% and 45.67%, 48.21%, respectively, compared to the pathogen control plants (Figure 4b,c). PAL activity was increased up to 44.12% (NPs 150 ppm + Pathogen) and 39.87% (NPs 200 ppm + Pathogen) in a similar scenario (Figure 4d).

### 3.5 Effect of titanium dioxide nanoparticles on the growth parameters of sorghum

**FIGURE 5:**
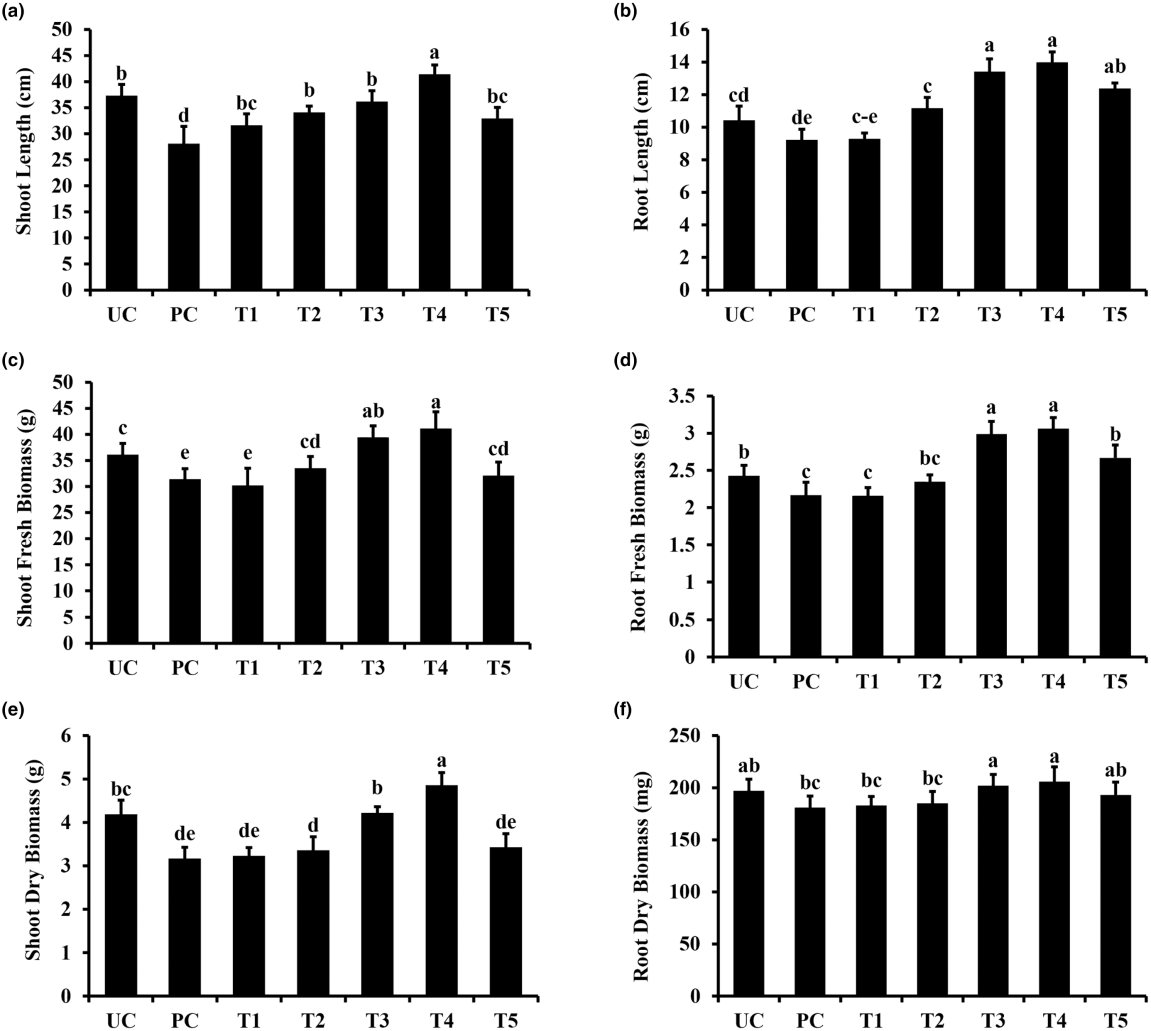
Effect of titanium dioxide NPs on growth attributes of Sorghum Plants. (a) Shoot Length, (b) Root Length, (c) Shoot Fresh Biomass, (d) Root Fresh Biomass, (e) Shhot Dry Biomass, (f) Root Dry Biomass. Vertical bars represent the standard error. Small letters represent the level of significance according to ANOVA and DNMRT (p 〉 .05). PC, pathogen alone; T1, TiO2 NPs @ 50 ppm + Pathogen; T2, TiO2 NPs @ 100 ppm + Pathogen; T3, TiO2 NPs @ 150 ppm + Pathogen; T4, TiO2 NPs @ 200 ppm + Pathogen; T5, copper oxychloride @ 2.4 g/L + Pathogen; UC, untreated control.

Sorghum plants showed reduced growth when exposed to pathogens. Data presented in Figure 5 show the effects of pathogens and nanoparticles applied in different combinations on sorghum plant growth parameters, including shoot, root length, shoot, root fresh biomass, and shoot/root dry biomass. The shoot and root lengths of sorghum plants were analyzed at 30 days post‐emergence. As can be seen in Figure 5, the shoot and root length decreased significantly in pathogen‐treated plants, where the presence of nanoparticles increased the shoot and root length in a dose‐dependent manner. The positive effects of NPs on shoot length were more evident at a concentration of 200 ppm, where an increase of 61.34% and 49.26% was noted in the presence (PC) and absence of pathogen (UC), respectively (Figure 5a).

In a similar scenario, the root length in sorghum plants treated with TiO_2_ NP (200 ppm) showed a significant increase of 23.05% and 30.81%, respectively, as compared to the non‐treated control (UC) and pathogen control (PC) treatments (Figure 5b). Likewise, an increase in fresh biomass was observed in sorghum plants when treated with NPs (Figure 5c,d). Plants receiving NPs at 200 ppm concentration showed a significant increase of 19.17% (shoots) and 27.36% (roots) in the fresh biomass compared to non‐treated plants, respectively (Figure 5c,d).

Correspondingly, for the application of NPs, the observed increase in dry biomass accounted to 08.14% (shoots) and 13.78% (roots) for the 200 ppm concentrations compared to the non‐treated control, respectively (Figure 5e,f). The application of NPs (150, 200 ppm) elicited a similar positive response for the increase in dry biomass in the pathogen control (PC) treatment (Figure 5e,f).

### 3.6 Effect of titanium dioxide nanoparticles on yield parameters of sorghum

The applications of NPs or pathogens alone or in combination caused a significant effect on yield related traits of sorghum. The presence of the pathogen significantly (*p* < .05) decreased penical length (−17.38%) and the number of grains (−9.61%) in sorghum plants compared with the untreated control plants (Table 2). Whereas, the size of the panicle and the number of grains were increased in sorghum plants treated with NPs. The length of the panicle in sorghum plants treated with 150 and 200 ppm NPs increased up to 11.31% and 17.85%, respectively, as compared to the non‐treated control (Table 2). Likewise, the application of NPs at 150 and 200 ppm concentrations increased the number of grains up to 8.77% and 20.91% compared to the non‐treated control plants (Table 2), whereas the number of grains increased up to 20.83% (150 ppm) and 32.14% (200 ppm) compared to the pathogen control (PC) treatment (Table 2). In a similar scenario, the weight of grains per panicle was increased up to 14.69% (150 ppm) and 19.25% (200 ppm) in sorghum plants compared to the pathogen control (PC) treatment (Table 2).

**TABLE 2:** Effect of different treatments of TiO_2_ nanoparticles on yield‐related traits of sorghum plants.

Treatments	Length of panicles (cm)	Number of grains	Grain weight per panicle (g)
NC	23.61 ± 1.78^bc^	52.31 ± 3.27^cd^	63.04 ± 4.82^cd^
PC	19.36 ± 0.99^e^	47.52 ± 2.55^e^	59.03 ± 3.15^e^
T1	22.16 ± 1.26^b–d^	54.25 ± 4.81^b–d^	67.27 ± 4.01^c^
T2	24.37 ± 2.14^b^	59.21 ± 3.72^b^	72.39 ± 5.29^b^
T3	24.93 ± 1.82^b^	57.26 ± 4.06^b^	73.94 ± 4.62^b^
T4	28.42 ± 2.07^a^	65.12 ± 5.71^a^	79.23 ± 3.74^a^
T5	23.04 ± 1.51^bc^	56.13 ± 4.60^bc^	66.08 ± 4.57^c^

*Note*: Values are presented as mean ± standard error. Small letters represent the level of significance according to ANOVA and DNMRT (*p* > .05).

### 3.7 Analysis of the physiological parameters

**FIGURE 6:**
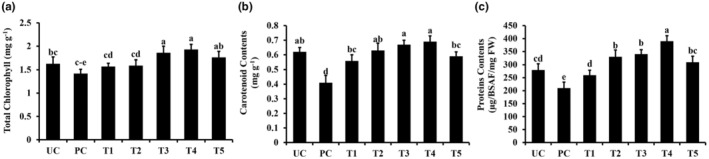
Effect of titanium dioxide NPs on the physiological attributes of Sorghum plants. (a) Total chlorophyll contents, (b) carotenoid contents, and (c) protein contents. Vertical bars represent the standard error. Small letters represent the level of significance according to ANOVA and DNMRT (*p* > .05). PC, pathogen alone; T1, TiO_2_ NPs @ 50 ppm + Pathogen; T2, TiO_2_ NPs @ 100 ppm + Pathogen; T3, TiO_2_ NPs @ 150 ppm + Pathogen; T4, TiO_2_ NPs @ 200 ppm + Pathogen; T5, copper oxychloride @ 2.4 g/L + Pathogen; UC, untreated control.

Parameters like total chlorophyll contents, carotenoids, and protein contents were decreased by pathogen inoculation compared to control plants (Figure 6). However, TiO_2_ NPs significantly improved total chlorophyll contents, carotenoids, and protein contents in pathogen‐inoculated plants (Figure 6). Treatments T3 (pathogen and TiO_2_ @ 150 ppm) and T4 (pathogen and TiO_2_ @ 200 ppm) significantly increased the total chlorophyll and carotenoid contents, even greater than untreated control plants (Figure 6a,b). TiO_2_ NPs were also effective in improving the total protein contents. Pathogen alone decreased total protein contents (Figure 6c). Whereas, the application of TiO_2_ NPs following pathogen contents significantly increased total protein contents compared to the pathogen alone and non‐treated control plants (Figure 6c).

### 3.8 Correlation and PCA analysis

**FIGURE 7:**
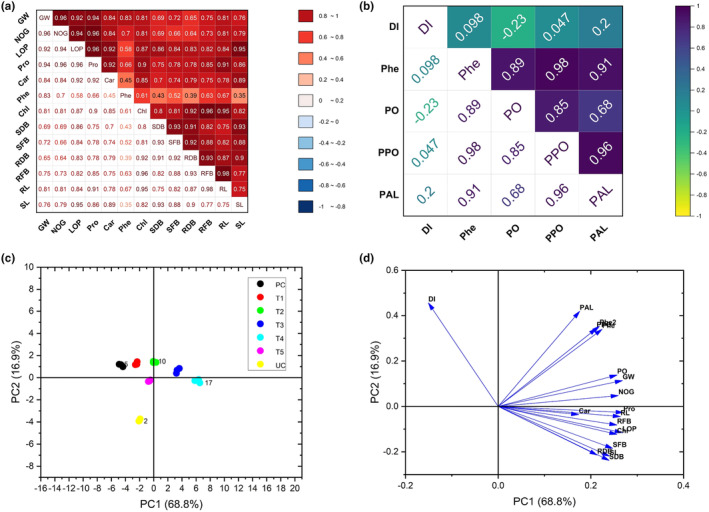
Matrix of correlation coefficients between growth and yield attributes (a) and disease and defense‐related attributes (b) of sorghum treated by TiO_2_ nanoparticles and *Colletotrichum graminicola*. Principal component analysis among different treatments (c) and biplot of investigated traits in sorghum plants (d) treated by TiO_2_ nanoparticles and *C. graminicola*. Car, carotenoid contents; Chl, total chlorophyll contents; DI, disease index; PAL, phenyl alanine ammonia lyase activity; PC, pathogen alone; Phe, total phenolic contents; PO, peroxidase activity; PPO, polyphenol activity; Pro, protein contents; RDB, root dry biomass; RFB, root fresh biomass; RL, root length; SDB, shoot dry biomass; SFB, shoot fresh biomass; SL, shoot length; T1, TiO_2_ NPs @ 50 ppm + Pathogen; T2, TiO_2_ NPs @ 100 ppm + Pathogen; T3, TiO_2_ NPs @ 150 ppm + Pathogen; T4, TiO_2_ NPs @ 200 ppm + Pathogen; T5, copper oxychloride @ 2.4 g/L + Pathogen; UC, untreated control.

The obtained data were further analyzed by performing a correlation matrix (Figure 7a,b) and PCA biplot analysis (Figure 7c,d). The results of correlation matrix analysis indicated a significant positive correlation between growth and yield‐related traits of sorghum plants (Figure 7a), whereas a negative relationship between disease index and accumulation of defense‐related compounds was seen (Figure 7b).

The principal component separated all the treatments into separate groups, inferring that these treatments were different from the control (Figure 7c). Nearly all the defense‐related and growth traits of sorghum plants were grouped as shown by PCA biplot analysis (Figure 7d). This presents their tendency to ameliorate the growth of the plants under pathogen stress (Figure 7d).

## 4 DISCUSSION

Recently, nanomaterials have been developed that could enhance the physiological systems of plants (Aqeel et al., 2022). Nanomaterials have multiple plant beneficial properties, such as growth promotion, induction of system resistance, and the ability to suppress the growth of plant pathogens (Servin et al., 2015). Nanoparticles (NPs) can be utilized in crop protection due to their high chemical and thermal endurance and inexpensive manufacturing process (Pramanik et al., 2023). The green‐fabrication of metal nanoparticles has numerous advantages over the conventional chemical methods of nanosynthesis. The phytoreduction of the compounds leads to the synthesis of biologically valuable nanomaterials with many biological applications (Husen & Siddiqi, 2014).

The phytochemical analysis of the pomegranate peel has shown that it includes a variety of distinct substances (Akhtar et al., 2015). Catechin and gallic acid were the next most prevalent phenolic components in the extract after chlorogenic acid. It was found that the primary phenolic component of pomegranate fruit, chlorogenic acid, was present in evenly distributed levels. The ongoing study reports the green synthesis of TiO_2_ NPs using pomegranate peel extract. The change in the color of the reaction mixture from milky white to pink‐brown was considered as an initial sign of synthesis. The synthesis of TiO_2_ NPs was further confirmed by measuring the absorbance using a spectrophotometer and observation of the surface plasmon resonance band in the range of 250–280 nm. This is due to the fact that TiO_2_ NPs show responses in the range of a particular wavelength. Our findings are in accordance with the published studies (Keßler et al., 2019; Santhoshkumar et al., 2014).

The physiochemical characterization of TiO_2_ nanoparticles was performed by different analytical methods. X‐ray diffraction (XRD) analysis was performed to investigate the crystallographic structure of the nanoparticles. The generated XRD patterns indicated unique diffraction peaks at 25.3, 27.5, 36.1, 41.2, 54.3, 56.6, etc., allowing the crystalline phases present in the sample to be determined. Fourier Transform Infrared Spectroscopy (FTIR) was performed to analyze the functional groups and chemical bonds present on the nanoparticle surface. The FTIR spectra displayed unique absorption bands, offering information on the chemical composition and surface changes of the nanoparticles (Huang et al., 2015). The shape and surface topography of the nanoparticles were visualized using SEM. The SEM scans revealed a variety of particle sizes and shapes, indicating the nanoparticles' effective production and dispersion.

TiO_2_ NPs have received considerable attention for their photocatalytic antimicrobial characteristics. The antimicrobial properties of TiO_2_ nanoparticles have been documented against different fungal and bacterial microbes (Subhapriya & Gomathipriya, 2018). The green‐synthesized TiO_2_ NPs demonstrated significant antifungal activity in this study. The antifungal activity of TiO_2_ NPs was assessed by a food poisoning technique against *C. graminicola*. A concentration dependent antifungal activity was observed. The application of TiO_2_ at concentrations of 150 and 200 ppm had the best antifungal effect. These outcomes were comparable with the fungicide control. In another study, TiO_2_ NPs showed antifungal activity against *Candida* spp. when used at concentrations of 256–512 ppm (Kermani et al., 2021).

A pot trial was performed to manage the anthracnose of sorghum using TiO_2_ NPs. The percent disease index was recorded against the disease after using NPs at different concentrations. A great variance in disease index was seen depending upon the concentration of NPs used. None of the concentrations used were effective enough to completely inhibit the disease development. However, NPs at 150 and 200 ppm effectively reduced the disease index by >60%. The highest disease index was given by the pathogen control treatment. Whereas, the lowest values for disease index were recorded in the sorghum plants treated with a 200‐ppm concentration of the green‐synthesized TiO_2_ NPs.

The large surface‐to‐volume ratio and reactivity have made TiO_2_ NPs effective antimicrobial agents (Amiri et al., 2022). Another probable antifungal mechanism of TiO_2_ NPs is the production of reactive oxygen species capable of damaging microbial cells (Ahmad et al., 2020), and the destabilization of the fungal outer covering, which alters the homeostasis of cells (Satti et al., 2021). The oxidation of the nuclear materials can occur with the entry of the TiO_2_ nanoparticles, leading to the disintegration of DNA and cell death (Satti et al., 2021). These could have attributed to the inhibition of fungal growth during in vitro analysis and the reduced disease index during the pot trials.

Phenolic compounds defend plants against invading pathogens. Pathogen stress increases the production of total phenolic contents in plants (Jiang et al., 2019). These compounds play an important role in maintaining plant growth and development under biotic constraints. Plants retain a biosynthetic pathway for the production of these phenolic compounds, which is driven by different enzymes like PO, PPO, and PAL (Siddiqui et al., 2009). Although the pathogen alone increased the production of total phenolics and the aforementioned defense‐related enzymes, the supply of TiO_2_ nanoparticles further enhanced the contents of total phenolics and the activities of enzymes in sorghum plants under pathogen stress. These results strongly establish the potential of green‐synthesized TiO_2_ nanoparticles in activating plant defense systems against pathogens. Different previous studies have also demonstrated that the exogenous application of nanoparticles increased the accumulation of total phenolics and the activity of defense‐related enzymes (Abdelrhim et al., 2021; Kumari et al., 2023). As was shown by the correlation coefficient values in our study, there were considerable negative correlations of the accumulation between defense‐related compounds and the disease index in sorghum plants.

The amelioratory effect of green‐synthesized TiO_2_ NPs on plant growth, yield, and physiological parameters was also studied. The onset of anthracnose disease significantly decreased growth and yield‐related attributes in sorghum plants inoculated with *C. graminicola*. The data showed that TiO_2_ NPs positively influenced the shoot, root length, and biomass as compared to the pathogen‐only treatment. The maximum growth indices were observed when the anthracnose infected sorghum plants were supplied with 150 and 200 ppm concentrations of green‐synthesized TiO_2_ nanoparticles. The findings also showed a significant increase in the chlorophyll, carotenoid, and total protein contents by the exogenous application of TiO_2_ nanoparticles.

Similarly, results revealed a negative impact of the anthracnose pathogen on the yield attributes of the sorghum plants. A significant decrease in spike length, grain weight, and grains per spike was seen in pathogen‐inoculated plants compared to the control plant. The significantly positive impact on the yield attribute of the sorghum plants was seed with the exogenous supply of green‐synthesized TiO_2_ nanoparticles on anthracnose‐infected sorghum plants. However, pronounced improvements were observed in plants given 150 and 200 ppm concentrations of TiO_2_ nanoparticles under *C. graminicola* infection. Moreover, the correlation matrix of the data on growth and yield‐related attributes represents a moderate to strong correlation between the variables. The same can be seen regarding each variable in the PCA biplot. These findings are in accordance with previous studies reporting that TiO_2_ nanoparticles significantly elevated the growth and yield attributes of wheat crops against Bipolaris leaf spot disease (Satti et al., 2021) and rust disease caused by *Puccinia striiformis* (Satti et al., 2022). The increase in growth and yield attributes can be due to the sequestration mechanism of nutritional elements by NPs (Mustafa et al., 2022). NPs increase the ability of plants to absorb and use water and fertilizers in a more efficient way (Iqbal et al., 2017). TiO_2_ NPs are reported to increase the *in‐planta* accumulation of different nutritional elements in the shoots of *Coriandrum sativum* plants (Gohari et al., 2020). Taken together, these beneficial aspects of nanoparticles aided in the increased growth and yield of sorghum plants under pathogen attack.

## 5 CONCLUSION

We focused on the green synthesis of TiO_2_ nanoparticles from the extract of pomegranate peel. The physiochemical analysis supported the synthesis of the required size and properties of the nanoparticles responsible for their stabilization and biological potential. The exogenous applications of these nanoparticles on sorghum plants caused a significant reduction in disease incidence and triggered the defense responses in sorghum plants. The findings confirm the great possibility of green‐synthesized TiO_2_ NPs in conventional agricultural products. The effectiveness of these NPs must be studied against numerous phytopathogens that significantly reduce agricultural yields in the field.

## AUTHOR CONTRIBUTIONS

**Ghulam Nabi:** Data curation (equal); formal analysis (equal); investigation (equal); methodology (equal); writing – original draft (equal); writing – review and editing (equal). **Tehmina Anjum:** Conceptualization (equal); data curation (equal); formal analysis (equal); resources (equal); supervision (equal); writing – original draft (equal); writing – review and editing (equal). **Zill‐e‐Huma Aftab:** Conceptualization (equal); data curation (equal); formal analysis (equal); resources (equal); supervision (equal); writing – original draft (equal); writing – review and editing (equal). **Humaira Rizwana:** Funding acquisition (equal); project administration (equal); writing – review and editing (equal). **Waheed Akram:** Conceptualization (equal); project administration (equal); resources (equal); software (equal); writing – original draft (equal); writing – review and editing (equal).

## FUNDING INFORMATION

This research was funded by Researchers Supporting Project number (RSPD2024R1048), King Saud University, Saudi Arabia.

## CONFLICT OF INTEREST STATEMENT

All the authors of this manuscript certify that they have no financial or other type of conflict of interest. The final manuscript of this work has been read and approved by all the authors.

## Data Availability

All the data supporting this study is contained in the manuscript.
